# Development of a comprehensive real-world evidence dataset to inform diabetes policy: the diabetes data cell project

**DOI:** 10.1186/s12889-026-27916-x

**Published:** 2026-06-02

**Authors:** A. Lavens, D. Jaminé, M. Urbina, B. Vaes, C. Mathieu, F. Nobels, the Steering committee of the Diabetes Data Cell Project, Margot Buyle, Thierry Moureaux, Philippe Lysy, Geert Goderis, Catherine Lucet

**Affiliations:** 1 Health Services Research, Sciensano, Brussels, Belgium; 2 Intermutualistic Agency, Brussels, Belgium; 3 Department of Public Health and Primary Care, KU Leuven, Leuven, Belgium; 4 Clinical and Experimental Endocrinology, University Hospitals Leuven—KUL, Leuven, Belgium; 5 Department of Endocrinology, AZORG, Aalst, Belgium

**Keywords:** Diabetes, Real-world data, National diabetes registry, Data linkage, Health administrative data, Non-communicable disease monitoring, Health policy

## Abstract

**Background:**

Diabetes is a highly prevalent non-communicable disease with a growing global burden, posing significant challenges to healthcare systems. Effective management requires comprehensive monitoring of prevalence, risk factors, and outcomes across diverse diabetes populations. While several European countries have established or are establishing national diabetes registries to guide care and policy, Belgium’s diabetes data landscape remains fragmented, lacking a unified national diabetes registry. Existing datasets cover limited populations and care settings, hindering a complete understanding of diabetes care and prevalence. To address this, the Belgian Diabetes Forum initiated the *Diabetes Data Cell Project*, aiming to integrate multiple healthcare datasets and create a comprehensive national diabetes registry.

**Methods:**

An inventory of existing databases was compiled, and the quality of the datasets was assessed based on predefined criteria. A selection of core datasets was made to ensure reliable identification of individuals with diabetes, based on clinical diagnoses and coverage of the entire healthcare spectrum. Complementary datasets were selected to enrich the core datasets with additional information on healthcare utilization and mortality. All datasets had to allow for linkage at the individual level and provide longitudinal data.

**Results:**

We identified two large core datasets of people with diabetes who are treated in primary care and in hospital outpatient clinics. We obtained permission from the Information Security Commission to link both sets for the period 2017–2027 at the individual level to the national insurance dataset — where all medical procedures are recorded. That insurance dataset is also linked to mortality data. The first data extraction, covering 2017–2024, resulted in a linked dataset of 76 964 unique individuals. The combined dataset provides a comprehensive overview of diabetes diagnosis and type, diabetes duration, demographic characteristics, clinical outcomes, laboratory results, medications and other treatments, medical procedures and hospitalizations, complications, healthcare utilization, mortality and costs of care.

**Conclusions:**

This study demonstrates that large-scale linkage of Belgian health data is feasible within the current legal and technical framework and enables the identification of people living with diabetes across both primary and specialized care. By integrating clinical and administrative datasets at the individual level, the project provides a comprehensive and longitudinal view of diabetes care, supporting analyses of treatment patterns, care processes, outcomes, and healthcare utilization. This work lays the foundation for a continuously updated, ‘*living’* national diabetes registry in Belgium and represents an important step towards more integrated, data-driven healthcare policy.

**Supplementary Information:**

The online version contains supplementary material available at 10.1186/s12889-026-27916-x.

## Background

Diabetes is a leading non-communicable disease (NCD), affecting an estimated 537 million adults worldwide in 2021 [1, 2]. Its increasing prevalence, together with population ageing and longer survival with chronic conditions, places growing demands on healthcare systems. Monitoring accurately disease burden, quality of care, and outcomes is essential to inform public health policy and healthcare planning. National diabetes registries support these objectives by enabling epidemiological research and evaluation of care quality [3–7]. However, developing such registries is challenging, as diabetes care spans multiple care providers and settings, requiring the integration of heterogeneous data sources.

**Fig. 1:**
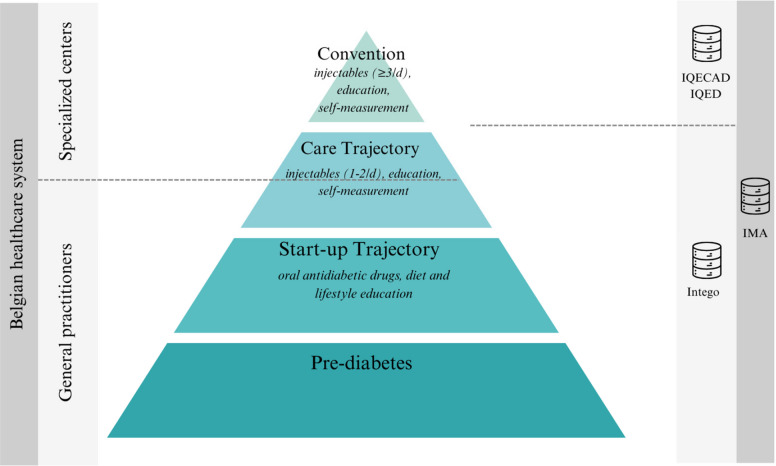
Overview of the structured diabetes care pathways in the Belgian healthcare system. The pyramid spans from pre-diabetes management in general practice to increasingly support in the Start-up Trajectory, the Care Trajectory (1–2 daily injectables) coordinated between general practitioners and specialized centers, and the Convention (≥ 3 daily injectables) within specialized centers. Belgian datasets (Intego, IMA, IQED, IQECAD) correspond to the respective levels of care shown on the right

In Belgium, diabetes care is delivered through different care pathways depending on disease severity and treatment needs (Fig. 1). People with type 2 diabetes managed with lifestyle changes, dietary advice, and oral medications are followed by their general practitioner (GP) and can enroll in the Start-up Trajectory. When treatment escalates to one or two daily insulin injections or Glucagon-like peptide-1 receptor agonists (GLP-1) injections, patients transition to the Care Trajectory, which involves coordinated care between the GP and an endocrinologist. Individuals with type 1 diabetes or advanced type 2 diabetes requiring intensive insulin therapy and managing complications, are treated by multidisciplinary teams in hospital-based diabetes polyclinics under the Diabetes Convention. For patients with complex foot problems, multidisciplinary specialized care is provided through the Diabetes Foot Convention in dedicated diabetes foot clinics.

Correspondingly, data collection is dispersed across several registries and datasets targeting specific subgroups, including patients in specialized diabetes centers, separately for pediatric [8] and adult care [9], and those with diabetic foot complications [10]. Data collection for shared care within the Belgian diabetes Care Trajectories is limited and sporadic [11]. No structured data collection exists for GP-based care in the Start-up Trajectory, although some Flemish GP practices voluntarily record patient data, including for diabetes [12, 13]. In addition, a registry captures genetic and immunologic data for newly diagnosed type 1 diabetes patients [14]. Finally, as in any country, administrative national datasets further provide information on reimbursed healthcare services (e.g. medication, laboratory tests, procedures, and surgery), as well as hospitalization and mortality data, but lack detailed clinical information so diabetes diagnosis relies on imperfect proxies [15]. As a result, diabetes data in Belgium are fragmented and not routinely linked across sources. This limits the ability to capture the full care trajectory of all individuals living with diabetes and to estimate the national burden of the disease, unlike in several other European countries where national diabetes registries enable comprehensive follow-up [16–19]. Although this gap has been recognized by the Belgian Diabetes Forum (BEDF), which brings together key stakeholders (including policy makers, care givers and patients) to define priorities for diabetes care, a comprehensive national diabetes registry is still lacking [20].

Recent European initiatives, including the European Health Data Space (EHDS) and the Joint Action on Cardiovascular Diseases and Diabetes (JACARDI), aim to facilitate the reuse and linkage of health data for research and policy purposes [21, 22]. These developments provide an opportunity to address existing data gaps.

This study aims to assess the feasibility of linking existing individual-level healthcare datasets in Belgium. By doing so, it provides a first step towards the development of a comprehensive national diabetes registry that is continuously updated (a ‘*living’* diabetes registry).

## Methods

### Inventory of datasets

An inventory of diabetes datasets in Belgium was compiled, building on and updating previous work [23]. This inventory was updated through consultations with experts from the various dataset holders. For each dataset, information was collected on hosting institution, data collection methods and frequency, population coverage and/or sampling, data content, and strengths and limitations of the dataset.

Dataset quality was assessed through discussions among the research team and the dataset owners using predefined criteria. *Accessibility* was defined as the feasibility of obtaining data access within a reasonable timeframe, considering legal and administrative procedures. *Continuity* referred to the availability of longitudinal data to assess trends over time. *Population coverage* described the extent to which the dataset represents the target population (e.g. national vs. regional, specific subgroups). *Reliability of diabetes diagnosis* was defined as the degree to which diabetes status was based on clinically validated diagnoses rather than proxy indicators. *Linkage potential* referred to the presence of unique identifiers enabling linkage with other datasets.

### Dataset selection

Datasets were categorized as *core* or *complementary*. Through discussions among the research team, core datasets were selected to ensure reliable identification of individuals with diabetes based on clinical diagnosis and coverage across the full spectrum of care. These datasets were required to:

- include clinically validated diabetes diagnosis;
- collectively cover primary and specialized care settings;
- provide longitudinal data;
- allow individual-level linkage; and
- contain key clinical variables (e.g. diagnosis, laboratory results, medication, comorbidities, and outcomes).

Complementary datasets were selected to enrich the core datasets and were required to:

- allow individual-level linkage;
- provide longitudinal data;
- contribute additional information on health status or healthcare utilization.

The time window 2017–2027 was chosen to include recent historical data, ensure relevance for current policy, and align with data availability and expected continuity of participating datasets.

### Data framework and linkage

In collaboration with the data managers and Data Protection Officers (DPOs) of the selected datasets, a theoretical framework was developed to define the overall objectives, the selected variables, and data flow.

Linkage at the individual patient level was enabled through the National Identification Number for Social Security (NISS), a unique identifier used for social security and healthcare purposes, including access to healthcare records and social security benefits such as insurance, pensions, and healthcare services. eHealth, the federal agency ensuring secure electronic information exchange, acted as a trusted third party (TTP) for pseudonymization.

### Data access and governance

In Belgium, accessing and linking existing datasets requires approval from the Social Security and Health Chamber of the Information Security Committee (ISC), the official national authority that proactively assesses compliance with the General Data Protection Regulation (GDPR) and sets the information security requirements for data exchange. The ISC request, including legal foundation, overall objectives, data flow and an overview of the selected variables along with their required justification, was prepared with the assistance of data managers and DPOs from the selected datasets. The legal basis for data processing in this study was identified as Article 9(2)(i) of the GDPR, which permits the processing of special categories of personal data (such as health data) when necessary for reasons of public interest in the area of public health. The data flow and Data Protection Impact Assessment (DPIA) were established in collaboration with the DPOs. In addition, a theoretical small cell risk analysis (SCRA) of the final dataset was initiated to ensure adequate protection of individual privacy.

A privacy statement for the *Diabetes Data Cell Project* has been created in order to inform patients about the re-use of their collected data, and is accessible online [24].

Procedures and timelines were documented to assess feasibility. Challenges related to legal constraints, data availability, and linkage processes were systematically recorded.

A steering committee was established, including representatives from dataset holders and the BEDF. The committee provided oversight throughout the project and is responsible for prioritization of research questions and publication policies.

## Results

### Selected datasets

An overview of the available diabetes-related datasets in Belgium, including their strengths and limitations for linkage, is presented in Table 1.

**Table 1:** Overview of relevant diabetes datasets in Belgium

Dataset (project) and type	Hosting institution	Period	Population	Content	Strengths	Limitations
Survey data						
Health Interview Survey (HIS) https://www.sciensano.be/en/projects/health-interview-survey	Sciensano	Repeated cross-sectional, every 4 years since 1997 (1997, 2001,2004,2008,2013,2018,2023) Face-to-face interviews and questionnaires	Representative sample of Belgian population (± 10 000 respondents) HIS2023: agreement to be recontacted. No NISS stored, but re-linkage to NISS possible via TTP (Statbel)	Medication use, health behavior (physical activity, diet, smoking, alcohol), BMI, self-reported information on chronic diseases	Representative for the population by making use of post-stratification weights Extensive lifestyle and sociodemographic info Linkage with NISS possible (via Statbel)	Low proportion of T2D patients (6,2%) Self-reported data, recall bias Selection and sample bias People living in institutions other than nursing homes (e.g. prison, psychiatric institution) are exluded. People living in nursing homes are included. If people cannot answer themselves a proxy interview is possible Time lag: data of 2018 was available in 2021, data collection 2023 delayed No clinical data No information about type of GP-practice Under-representation of people with lower social status
Belgian Health Examination study (BELHES) https://www.sciensano.be/en/projects/health-examination-survey	Sciensano	Cross sectional: 2018, as a secondary study of HIS2018	Representative subsample of the HIS (N = ± 1200 respondents, > = 18 years)	Blood and urine test, blood pressure measure, BMI (stored in Sciensano Biobank)	Clinical data Can be linked to HIS data (Statbel is TTP), legal framework/consent available Only source to determine the proportion of undiagnosed patients	Small sample (+ same limitations HIS, except for recall bias) Currently no framework to repeat it on a regular basis
Health Professional Survey data						
Sentinel Network of General Practitioners (SGP) https://www.sciensano.be/en/projects/network-general-practitioners	Sciensano	Longitudinal: since 1979 periodic modules to monitor one or more specific illness problems	A network of 100 GP practices (their patient population covers 1–1,5% of the Belgian population)	Morbidity in primary care, sociodemographic, diagnostic, treatment and healthcare data	Representative for Belgian GP workforce Able to study the evolution and epidemiology of certain diseases	Quality of data strongly depends on the reporting quality of GPs No NISS to link to other datasets The most recent T2D registration was in 2010 Currently diabetes not on the list of specific problems
EHR – primary healthcare						
Intego https://www.intego.be/over-intego	KU Leuven (ACGP)	Longitudinal: since 1999	Patient population of ± 130 participating GP practices Yearly contact group of about 350 000 patients, 18 000 with diabetes	Morbidity in primary care, diagnostic data, sociodemographic data, healthcare and medication data The data is aimed to perform audits of the primary care	Representative for Flemish population ± 5% of the national population A lot of diagnostic and clinical longitudinal data Large number of patients Linkage with NISS possible	Quality of data depends on coding behaviour of clinicians and there is a lot of variation therein between GPs Data from specialists as well as events that occur in hospital are not fully captured
Diabetes barometer https://www.intego.be/en/barometers	KU Leuven (ACGP)	Semiannual data collection, started in May 2023 Linked to the integrated general practice premium	At the moment about 4000 GP practices, this is expected to further increase Reporting via Healthstat portal from Healthdata.be	Indicators of quality of care given to people living with T2D and aged > 40 years	Automatic extraction from the electronic patient records Aimed for population management in GP practices, public health monitoring and research purposes, aiming to improve diabetes care and outcomes	Aggregated data No NISS to link to other datasets
Ambulatory Healthcare Information Laboratory (ACHIL) https://www.sciensano.be/en/biblio/de-zorgtrajecten-diabetes-mellitus-type-2-en-chronische-nierinsufficientie-impact-op-de-kwaliteit	Sciensano	Cross sectional, only once (2009–2013) Care Trajectory patients	Data of 2009–2011 Combination of data from 4 pillars: 1. GP via web application 2. IMA 3. Intego 4. SGP	Parameters by pillar: 1. Processes and outcomes of HbA1c, LDL-cholesterol, weight, height and blood pressure 2. Processes, healthcare utilization and costs 3. Demographic data, laboratory and clinical data, information on complications and comorbidities, and treatment 4. Processes, outcomes, and reasons why the patient has not started a care pathway for patients eligible for the care pathway	Nationwide data collection First evaluation of the effectiveness of care pathways to improve the quality of care in the field	Limited set of variables No longitudinal follow up No NISS to link to other datasets
Evaluation of Ambulatory Care Quality (EVACQ) https://www.sciensano.be/nl/biblio/de-zorgtrajecten-diabetes-mellitus-type-2-en-chronische-nierinsufficientie-en-kwaliteit-van-zorg	Sciensano	Cross sectional, only once (2017–2019) Care Trajectory patients	Data of 2009–2016 Combination of data from 2 pillars: 1. GP via webapplication (HD4PrC) 2. IMA	1. Processes and outcomes of HbA1c, LDL-cholesterol, weight, height and blood pressure 2. Processes, healthcare utilization and costs	Nationwide data collection Second evaluation of the effectiveness of care pathways to improve the quality of care in the field	Limited set of variables No longitudinal follow up No NISS to link to other datasets
Register data from hospitals						
Belgian Diabetes Register http://www.bdronline.be/index.php?id=55&sid=55&taal=E&mnav=1	Belgian Diabetes Registry Foundation	Longitudinal: since 1989	New patients < 40 years old diagnosed with T1D	Sociodemographics, clinical data	Clinical data Longitudinal	Only T1D patients No NISS to link to other datasets
Initiative for Quality improvement and Epidemiology in Diabetes (IQED) https://www.sciensano.be/en/projects/initiative-quality-improvement-and-epidemiology-diabetes	Sciensano	Biannial, repeated cross-sectional, since 2001	All Belgian specialized diabetes centers Obligated participation in the context of the diabetes convention Representative sample of adults (≥ 16 years) (10%)	Clinical hospital data, sociodemographic, diabetes type, examinations, complications, treatment, lab data and test results	NISS as unique identifier, allows linkage to other datasets Linkage established with national registry for vital status Clinical data Focus on quality indicators	Focus on patients treated in the diabetes convention (= on intensive insulin regimen) All T1D T2D on 3 or more insulin injections a day
Initiative for quality improvement and epidemiology among children and adolescents with diabetes (IQECAD) https://www.sciensano.be/en/biblio/initiative-quality-improvement-and-epidemiology-among-children-and-adolescents-diabetes-iqecad	Sciensano	Biannial, repeated cross-sectional, since 2008	All Belgian specialized diabetes centers Obligated participation in the context of the diabetes convention for children and adolescents All children and adolescents (0–19 years) with diabetes, 100% sample	Clinical hospital data, sociodemographic, diabetes type, examinations, complications, treatment, lab data and test results	NISS as unique identifier, allows linkage to other datasets Linkage established with national registry for viral status Clinical data Focus on quality indicators All children with diabetes	/
Initiative for Quality improvement and Epidemiology in multidisciplinary Diabetic Foot Clinics (IQEDFOOT) https://www.sciensano.be/en/projects/initiative-quality-improvement-and-epidemiology-multidisciplinary-diabetic-foot-clinics	Sciensano	Biannial, repeated cross-sectional, since 2005	All Belgian specialized foot clinics Obligated participation in the context of the diabetic foot convention	Clinical hospital data, sociodemographic, diabetes type, examinations, complications, treatment, lab data and test results	NISS as unique identifier, allows linkage to other datasets Linkage established with national registry for viral status Clinical data Focus on quality indicators	Only first 52 patients of each clinic presenting a foot problem with wagner grade classification 2 or more
Administrative data						
Intermutualistic Agency, Pharmanet https://aim-ima.be/?lang=nl	IMA	Gathered from the seven Belgian health insurance funds that manage compulsory health insurance Longitudinal since 2002 EPS available of ± 300 000 pts	National, entire ensured population (> 99%) Billing data collected by all illness funds	Sociodemographic data, healthcare and costs data, medication data (all reimbursed medication and health services), hospital visits (duration)	Inexpensive compared to original data collections General population data Detailed healthcare data Continuously collected Standardized data registration Linkage based on a unique identintifier (NISS)	Not collected and designed for scientific purposes: not structured in readily available variables for analyses Lack of clinical and diagnostic information No information about health behavior, BMI, etc Time lag: data is available in February year X of Year X – 2

Three core datasets and one complementary dataset were selected for linkage.

The selected core datasets were:

- The Integrated Computerized Network of General Practitioners (Intego) from the KU Leuven (Academic Centre of General Practice (ACGP)) as representative dataset of people living with diabetes followed in primary care [12, 13, 15].
- The Initiative for Quality improvement and Epidemiology in Children and Adolescents with Diabetes (IQECAD) and the Initiative for Quality improvement and Epidemiology in Diabetes (IQED) from Sciensano as representative datasets of people living with diabetes, treated with intensive insulin therapy and followed in specialized care settings for children and adolescents, or adults respectively [9, 25, 26].

The complementary dataset was:

- Intermutualistic Agency (IMA) as national combined dataset of all seven health insurance funds, to enrich the core datasets with data on use of medicines, consultations and medical procedures [27]. This dataset also provides detailed costs of care, and contains information on mortality (vital status and date of death) through linkage to the national population registry.

More details about the datasets can be found in additional file 1.

### Data flow and selected data

**Fig. 2:**
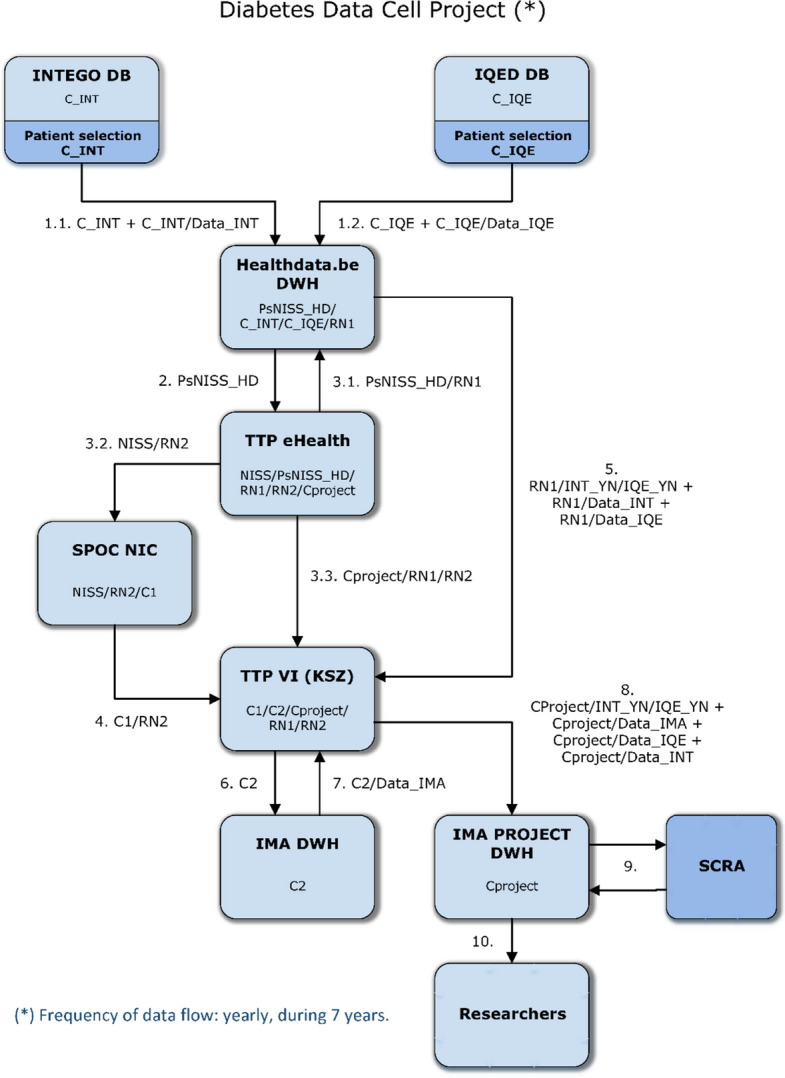
Schematic overview of the data flow. A concise explanation of the data flow is given in additional file 1

The data flow and stepwise procedure are presented in Fig. 2 and explained in the additional file 2. The study included all individuals registered during 2017–2027 in the IQECAD and IQED datasets, as well as all individuals registered during 2017–2027 in Intego who have been diagnosed with diabetes. For detailed information on the identification of people living with diabetes in the selected datasets, see additional file 1. All relevant data, including each patient’s available historical information (prior to 2017), were extracted for the selected individuals. The selected variables per dataset are included in Additional file 3.

Contents of the combined dataset include: diabetes diagnosis and type, diabetes duration, demographic characteristics (age, sex, place of residence, assisted living), clinical outcomes (blood pressure, weight, height, body mass index, results of examinations), laboratory results (glycemic measures, lipid profiles, renal function, etc.), medications (prescriptions, posology) and other treatments, medical procedures and hospitalizations, complications (largely inferred from treatments), healthcare utilization (which providers are involved, frequency of visits, care paths), mortality and costs of care.

The IMA data warehouse was selected to host the linked dataset. Only researchers of the *Diabetes Data Cell Project* can access the combined dataset using a Virtual Private Network (VPN), after the results of the SCRA are applied. The following restrictions for the combined dataset were identified based on the SCRA:

- Aggregation of all location-related information to the level of the administrative district (arrondissement);
- Aggregation of country-related information to prevent the occurrence of small cells;
- Conversion of all relative dates into true relative dates, using a fictive birth date;
- Limiting the number of healthcare provider service codes and professional qualifications to the information strictly necessary for the study, either by grouping less frequent categories into an “other” class or by aggregating detailed codes into broader categories.

The data flow was designed to be executed once a year, starting from the available part of the study period following approval of the study, and intended to be repeated until the complete study period (2017–2027) was included (maximum 7 times). From the second execution of the data flow onwards, data from both the newly added study years and the previously included study years will be transferred to capture any corrections made to the individual datasets. The combined dataset will be available until 5 years after having received the last data (estimated 2034). External parties that would like to access the combined dataset are required to submit a request through the Health Data Agency (HDA) (https://www.hda.belgium.be/en/data_request). An approval from the ISC will be required.

### Implementation and feasibility

At the end of 2022, the *Diabetes Data Cell Project* was discussed with the selected dataset holders (Sciensano, KU Leuven (ACGP), and IMA), who agreed to collaborate on this project. This project further got the support of the Belgian Ministry of Health as first use case of the HDA.

The deliberation was submitted on March 13 and issued on May 17, 2024. The ISC approved the study (nr 24/062), and the deliberation remains valid for 10 years.

**Fig. 3:**
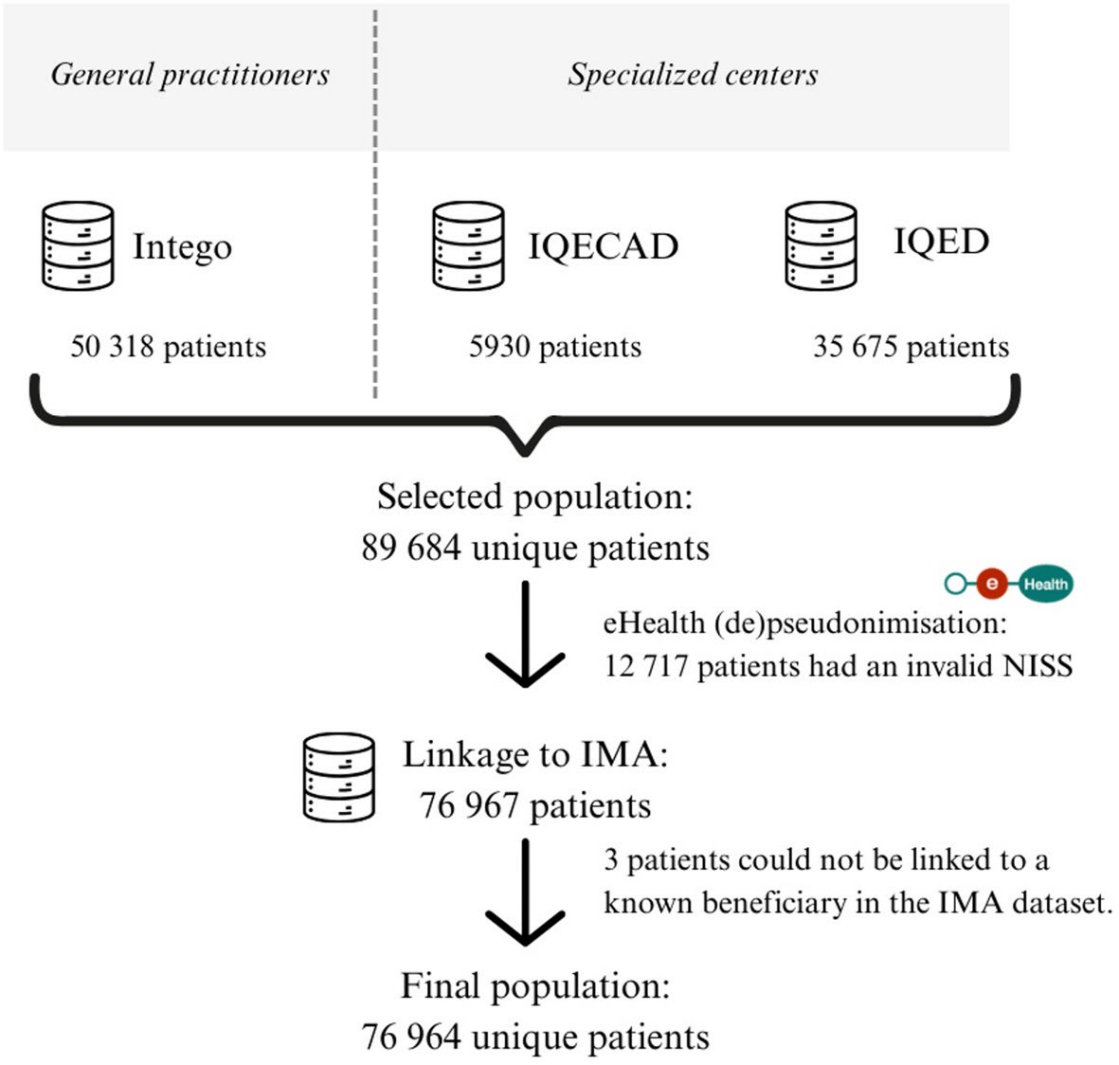
Flowchart of the selection of individuals living with diabetes (data 2017–204). The diagram outlines the identification of eligible individuals from general practitioner and specialized center datasets, the application of the eHealth (de)pseudonimisation process, linkage to the IMA dataset, and the final study population

**Fig. 4:**
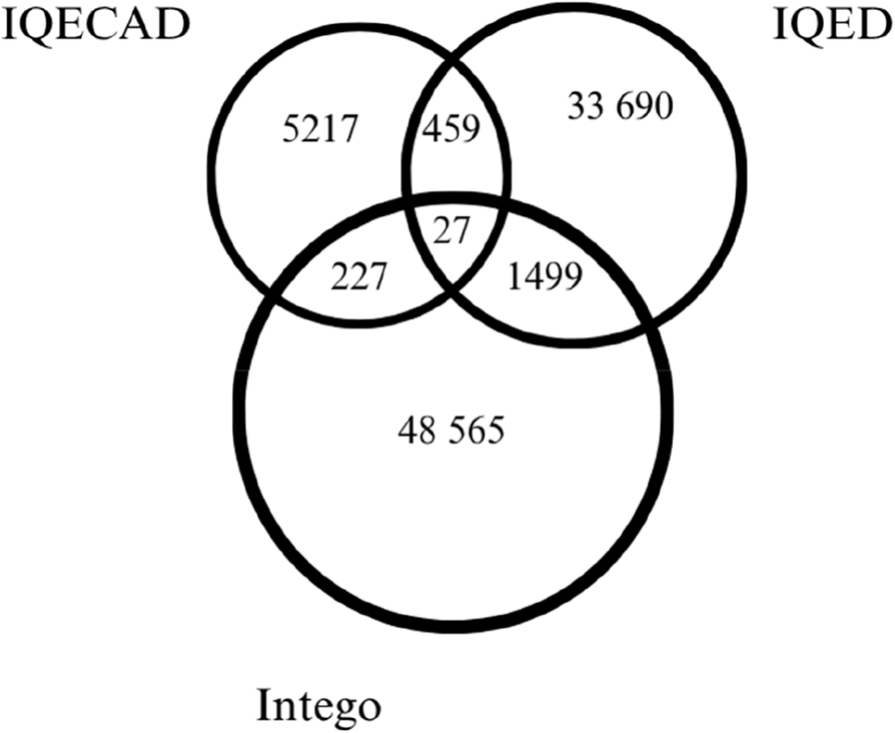
Schematic overview of the selection of individuals per dataset and overlap between the different datasets (data 2017–2024)

The first selection of people living with diabetes and their clinical data took place in June 2025, and included data from those being registered in Intego, IQECAD or IQED between 1 Jan 2017 and 31 December 2024. The number of selected individuals in the individual datasets as well as in the combined dataset are presented in Fig. 3. Of the 50 318 individuals identified in Intego, 1499 were also included in IQED, 227 were also present in IQECAD, and 27 individuals appeared in all three datasets. Between IQED and IQECAD, an overlap of 459 individuals was found (Fig. 4). Due to an invalid NISS, linkage could not be performed for 5615 individuals identified in Intego, 7102 individuals identified in IQED and IQECAD, and 247 individuals included across all three datasets. The final dataset, after the exclusion of 3 individuals that could not be linked to a known beneficiary in the IMA dataset, included 76 964 individuals (Fig. 3). As of today, the SCRA restrictions are not yet applied, and so the final dataset not accessible.

## Discussion

This project demonstrates the feasibility of linking multiple Belgian health datasets to construct a comprehensive national-level diabetes data cell. The resulting dataset integrates information across primary care, specialized care, and administrative sources, enabling detailed analysis of patient trajectories across the healthcare system.

Diabetes care in Belgium is tracked in several individual datasets and quality assurance initiatives, each with its own purpose. In our *Diabetes Data Cell Project*, data are linked at the individual patient level, enabling us to follow each patient’s journey across the different healthcare settings. This level of detail goes beyond what can be achieved with aggregated data, which cannot capture transitions between datasets or care settings, nor provide insights into individual quality of care or patient-specific risks of developing complications.

Because of the burden associated with data collection, the clinical core datasets used in this project register a limited number of parameters and include only representative samples of people living with diabetes, rather than the full population. We have been able to extensively increase the number of parameters through linkage with the complementary IMA dataset, that registers all use of medicines, consultations, medical procedures, costs and mortality. Ideally, these datasets would cover all individuals with diabetes, which is particularly important for adequately representing minority groups. We capture very well people with type 1 diabetes and type 2 diabetes on intensive insulin therapy through the IQECAD and IQED quality assurance initiatives under the framework of the diabetes convention. Because insulin delivery systems and self-management tools are reimbursed only through this diabetes convention, largely all are included in this convention. IQECAD collects data on all patients, but to reduce the data collection burden, IQED currently only includes a random 10% sample of patients. People with type 2 diabetes not on intensive insulin therapy are managed by their GP, and are not always enrolled in structured care programs. While Intego provides comprehensive primary care data extracted directly from electronic health records, it depends on accurate GP recording and may underestimate cases.

Since health insurance is obligatory in Belgium, the complementary IMA dataset could provide data of all Belgian people with diabetes, but it contains no data on diagnoses. We could rely on proxies to estimate diabetes, such as participation in diabetes care programs or the use of glucose-lowering medications, but there is a high risk of misclassification due to the increasing use of these medications for non-diabetic indications (e.g., GLP-1 receptor agonists for obesity and Sodium-Glucose Co-Transporter 2 (SGLT2) inhibitors for heart failure and renal insufficiency). The diabetes barometer project of the ACHG at KU Leuven aims to increase accurate identification of people living with diabetes among GPs [28]. The barometer project uses software to flag individuals with diabetes within GPs records. The project is supported by the National Institute for Health and Disability Insurance (NIHDI), and GPs receive remuneration for their participation. Actually 11 406 GPs across 4038 GP practices in Belgium are participating. This barometer project will further increase the accuracy of diabetes diagnosis in the Intego and IMA datasets. Further improvements in data collection – through measures such as better structuring and automating data extraction – could increase the IQED sample size. At the same time, providing incentives for GPs and/or reducing the administrative burden associated with registering patients in care pathways could help increase the national coverage of primary care datasets.

Further work is needed to implement the SCRA and finalize the combined dataset, ensuring data completeness, harmonization, and assessment of reliability and representativeness across the diabetes care continuum. Once established, the combined dataset will provide a valuable resource to address key research and policy questions, including the quality of diabetes care, medication adherence, complications, comorbidities, and identification of high-risk groups. By integrating longitudinal data, including available patient history prior to 2017, it will enable comprehensive analyses of care trajectories and outcomes over a follow-up period of up to 10 years, as well as the evaluation of clinical indicators and effects of changes in policy or reimbursement practices [29]. In future work, these questions will be defined and prioritized.

So far, we have already identified several barriers to the development of a *‘living’* diabetes registry. The main hurdle of this project lies in the current legal framework governing the linkage of health datasets, which is rigid and does not allow for the timely addition of new variables to the combined dataset. However, accurate and up-to-date assessments of quality of care require the inclusion of emerging treatments and technologies, such as novel glucose monitoring systems and hybrid closed-loop therapy. Although the core datasets are legally authorized and technically capable of incorporating such innovations for primary data use, this does not automatically extend to secondary data use. Consequently, adding new variables to create a *‘living’* registry requires an amendment to the original ISC approval, triggering a re-approval process.

Setting up and maintaining the data flow also requires significant resources. The existing infrastructure is struggling to meet the growing demand for secondary use of health data, resulting in discussions on prioritization, limited capacity, and delays in data availability. Although project discussions began in 2022, the first data flow was not established until mid-2025, and data access remains pending. This underscores the need for sustained political commitment and resource allocation to support such initiatives.

A relatively high proportion of individuals could not be linked due to technical issues in the secure reading and (de-)encryption of the NISS across different data partners. However, this issue could not yet be fully analyzed or resolved, again highlighting the need for a robust data flow, adequate resources, and appropriate technical solutions to address such challenges in a timely manner.

Belgium is not alone in facing challenges when developing a national diabetes registry, and valuable lessons can be drawn from countries like Scotland and Sweden where such registries are already established. The European Diabetes Forum (EUDF) has outlined key barriers and recommendations for advancing diabetes registries, highlighting legal issues such as data protection, governance, and limitations in data linkage [18, 19]. A review of multiple European diabetes registries further revealed considerable heterogeneity in governance structures, data infrastructure, coverage, and data periodicity [16]. Additionally, the GDPR and its varying interpretations across member states posed – until now—significant obstacles to cross-border or secondary use and linkage of health data [30]. Although a lot of these barriers will be handled by the upcoming EHDS regulation, ensuring that diabetes registries remain high on the political agenda will continue to be a key priority.

## Conclusions

This study demonstrates that large-scale linkage of Belgian health data is feasible within the current legal and technical framework and provides a unique opportunity to examine diabetes care pathways across sectors and over time. By integrating clinical registries and administrative databases at the individual level, the *Diabetes Data Cell Project* successfully identified people living with diabetes across the healthcare system. The resulting dataset supports robust investigation of medication use, care processes, complications, and comorbidities, while enabling longitudinal analyses of patient trajectories, risk groups, treatment patterns, and policy impacts. This approach establishes a strong foundation for a continuously updated, ‘*living*’ national diabetes registry and represents an important step toward more integrated health data use in Belgium. The framework is scalable and transferable, offering a model for other European countries and for broader application to non-communicable diseases.

## Supplementary Information

Additional file 1: Selected data sets

Additional file 2: Concise explanation of the data flow

Additional file 3: The selected variables per dataset

## Data Availability

No datasets were generated or analysed during the current study.

## Abbreviations

ACGP: Academic Centre of General Practice
BEDF: Belgian Diabetes Forum
DPIA: Data Protection Impact Assessment
DPO: Data Protection Officer
EHDS: European Health Data Space
EUDF: European Diabetes Forum
GDPR: General Data Protection Regulation
GLP-1: Glucagon-Like Peptide-1
GP: General Practitioner
HaDEA: European Health and Digital Executive Agency
HDA: Health Data Agency
IMA: Intermutualistic Agency
Intego: Integrated Computerized Network General Practitioners
IQECAD: Initiative for Quality Improvement and Epidemiology among Children and Adolescentswith Diabetes
IQED: Initiative for Quality Improvement and Epidemiology in Diabetes
ISC: Information Security Committee
ICPC: International Classification of Primary Care
JACARDI: Joint Action on Cardiovascular Diseases and Diabetes
NCD: Non-Communicable Disease
NIHDI: National Institute for Health and Disability Insurance
NISS: National Identification Number for Social Security
SCRA: Small Cell Risk Analysis
SGLT2: Sodium-Glucose Co-Transporter 2
TTP: Trusted Third Party
VPN: Virtual Private Network
