# Supplementary material for: Development of a comprehensive real-world evidence dataset to inform diabetes policy: the diabetes data cell project
Source: BMC Public Health. 2026 Jun 2;26:2286. doi: 10.1186/s12889-026-27916-x (PMC13445860; doi:10.1186/s12889-026-27916-x)
Supplement: Supplementary file 1 [file 12889_2026_27916_MOESM1_ESM.docx]

**Additional file 1 : Selected datasets**

**Intego**

Intego is a project of the ACGP at KU Leuven, which has been recording coded health-related personal data since 1994. Intego collects (fully automatically since 2004) comprehensive data from primary care, including physical examination findings, precise laboratory results, comorbidity, diagnostic information, sociodemographic data, and medication records. This dataset is designed to support audits and quality assessments of primary care practices. Currently, data from the electronic health records of approximately 350 000 individuals (the annual contact group), including about 18 000 people with diabetes, are automatically collected on a weekly basis from around 130 GP practices distributed across Flanders and made available to researchers. In 2017, the scope of the Intego database was expanded to mapping the impact of environment on public health. People living with diabetes registered in Intego are identified based on the International Classification of Primary Care (ICPC) codes assigned by the GP (T89; T90; W85) and/or registration of follow-up within integrated, multidisciplinary diabetes care pathways. The dataset is hosted at Healthdata.be at Sciensano.

**Initiative for Quality improvement and Epidemiology in Children and Adolescents with Diabetes (IQECAD) and Initiative for Quality improvement and Epidemiology in Diabetes (IQED)**

IQECAD and IQED are two quality improvement initiatives commissioned by the National Institute for Health and Disability Insurance (NIHDI) and conducted by Sciensano, for which every two years data is collected concerning demographics, anthropometry, precise laboratory results, comorbidities, treatment, and care outcomes of children and adults with diabetes who are followed in specialized diabetes centers under the framework of the diabetes conventions. Currently, data are collected from 17 pediatric and 100 adult diabetes centers in Belgium, in which approximately 3500 children and adolescents and 35 000 adults living with type 1 diabetes, and approximately 70 000 adults living with type 2 diabetes on intensive insulin therapy are followed. All specialized diabetes centers in Belgium are required to participate in these initiatives by registering 100% (IQECAD) or 10% (IQED, based on a variable and representative sample) of their patients. This makes IQECAD and IQED nationally representative databases of children and adults, respectively, with type 1 and type 2 diabetes who are followed in specialized centers. The core tasks of these initiatives are to map the prevalence of diabetes and its complications, and the progression of treatment and complications among people living with diabetes (the epidemiological component), as well as to measure and stimulate the quality of care provided within the framework of the convention through quality indicators (the quality improvement component). Every center receives an anonymized center-specific benchmarking feedback, and the general results are published in an anonymized global national report. All persons registered in IQECAD or IQED are diagnosed with diabetes and are therefore selected. These datasets are hosted at Healthdata.be at Sciensano.

**Intermutualistic Agency (IMA)**

The IMA dataset, which collects and centralizes data from all seven Belgian health insurance funds, including information on healthcare utilization (such as medical care and medication) and related costs, was chosen as a complementary dataset. Since all Belgian citizens (around 11 million) are required to have health insurance, the IMA dataset provides a comprehensive overview of the real-world use of healthcare procedures and services by all people across Belgium. The dataset includes data on outpatient care, hospitalizations, pharmaceutical prescriptions, mortality, and reimbursed medical procedures, enabling detailed analysis of healthcare utilization patterns and associated costs. For instance, it is possible to determine whether a person has received anti-VEGF eye injections or laser therapy, whether they are undergoing dialysis or have had a kidney transplant, which cardiac procedures have been performed, and whether foot or vascular surgery has taken place. However, as this administrative dataset does not include diagnostic information, diagnosis of diabetes requires the use of imperfect proxies. The dataset is hosted at IMA.
