# Supplementary material for: Development of a comprehensive real-world evidence dataset to inform diabetes policy: the diabetes data cell project
Source: BMC Public Health. 2026 Jun 2;26:2286. doi: 10.1186/s12889-026-27916-x (PMC13445860; doi:10.1186/s12889-026-27916-x)
Supplement: Supplementary file 2 [file 12889_2026_27916_MOESM2_ESM.docx]

**Additional file 2 : Concise explanation of the data flow**

Note: The data flow is executed once per year for a maximum duration of seven years.

Step 1: Initial Data Selection on the Healthdata Platform

1.1 The Intego database manager (hosted on the Healthdata.be platform) selects all relevant data (Data_INT) based on the project’s selection criteria (all diabetes patients from 2017 onwards), using the database-specific personal identifier (C_INT). A separate list of unique personal identifiers found in this dataset is prepared and transferred, along with the data, to the Healthdata.be administrator.

1.2 Similarly, the IQED/IQECAD database manager (IQED database, also hosted on Healthdata.be) selects all necessary data (Data_IQE) based on the same criteria, using a different personal identifier (C_IQE). A separate list of unique personal identifiers is also prepared and transferred to the Healthdata.be administrator.

Step 2: Data Processing on Healthdata.be

The Healthdata.be administrator replaces all database-specific personal identifiers (C_INT and C_IQE) in both datasets with a uniform, register-independent code (PsNISS_HD). Two new datasets are created: PsNISS_HD/Data_INT and PsNISS_HD/Data_IQE.

A consolidated list of unique PsNISS_HD identifiers is generated, indicating whether each identifier appears in the Intego or IQED databases: PsNISS_HD/INT_YN/IQE_YN.

The unique PsNISS_HDs of this list is then transmitted to the Trusted Third Party (TTP) eHealth for further processing. The list PsNISS_HD/INT_YN/IQE_YN and the datasets PsNISS_HD/Data_INT + PsNISS_HD/Data_IQE are stored for further processing in step 5.

Step 3: Anonymization by TTP eHealth

The TTP eHealth decodes each PsNISS_HD identifier into the corresponding social security number (INSZ).

A unique project-specific code (Cproject) is generated for each INSZ, along with two random numbers (RN1 and RN2).

Three conversion lists are created and distributed:

3.1. PsNISS_HD to RN1 (sent back to Healthdata.be).

3.2. INSZ to RN2 (sent to the National Intermutualistic College (NIC) contact point, the highest consultation body of the Belgian Health Insurance Funds).

3.3 Cproject to RN1/RN2 (sent to the Insurance Institutions’ TTP - Crossroads Bank for Social Security (TTP VI (KSZ)), reponsible for the elaboration of the E-government strategy within the Belgian social sector).

Step 4: Conversion at NIC

NIC converts the INSZ values in the INSZ/RN2 list to insurance institution codes (C1) and forwards the new C1/RN2 list to TTP VI (KSZ).

Step 5: Data Encoding at Healthdata.be

The Healthdata.be administrator replaces PsNISS_HD in the previously prepared datasets with the corresponding RN1.

The updated datasets (RN1/INT_YN/IQE_YN, RN1/Data_INT, and RN1/Data_IQE) are then transferred to TTP VI (KSZ).

Step 6: Insurance Institution Data Processing at TTP VI (KSZ)

TTP VI (KSZ) replaces C1 with the IMA insurance code (C2) and stores these codes in the IMA Data Warehouse (DWH).

Step 7: Data Extraction at IMA

IMA extracts all required data based on C2 (Data_IMA) and transfers C2/Data_IMA to TTP VI (KSZ).

Step 8: Final Anonymization at TTP VI (KSZ)

Using the step 3.3 conversion list, TTP VI (KSZ) replaces RN1 in the datasets from step 5 with the corresponding Cproject.

C2 values in the IMA dataset from step 7 are replaced with Cproject, ensuring full anonymization.

The final datasets (Cproject/INT_YN/IQE_YN, Cproject/Data_IMA, Cproject/Data_IQE, and Cproject/Data_INT) are transferred to the IMA Project DWH.

Step 9: Risk Analysis

A Small Cells Risk Analysis (SCRA) is conducted if required by the Information Security Committee (IVC).

Step 10: Data Access for Researchers

Once approved, the data is made available in the IMA Project DWH for research purposes.
