# Supplementary material for: Development of a comprehensive real-world evidence dataset to inform diabetes policy: the diabetes data cell project
Source: BMC Public Health. 2026 Jun 2;26:2286. doi: 10.1186/s12889-026-27916-x (PMC13445860; doi:10.1186/s12889-026-27916-x)
Supplement: Supplementary file 3 [file 12889_2026_27916_MOESM3_ESM.docx]

**Additional file 3: List of selected variables**

**APPENDIX LIST OF SELECTED VARIABLES IN IQED**

A detailed description of the dataset can be found online: <https://fair.healthdata.be/dataset/2e2adc57-1922-48f5-9d18-4a87ad7a8933>

All patients registered in IQED are diabetes patients and are selected as such. Based on the two personal lists drawn up from IQE(CA)D and Intego respectively, a third unique personal list is compiled which will be linked to IMA. In this new unique personal list, it will be indicated whether a patient is found in the IQE(CA)D database and/or the Intego database.

For the selected patients, the following data are required:

| **Variabele** | **Description** |
| --- | --- |
| IDC_PAT | Double-coded national registry number of the patient |
| DT_START_AUDIT | Start date of the IQED audit period |
| DT_END_AUDIT | End date of the IQED audit period |
| nr_path_birthy | Year of birth |
| nr_path_deathy | Year of death |
| CD_PAT_SEX | Sex (male/female/unknown) |
| CD_PAT_NIS | Place of residence of the patient, at district level |
| CD_CNTRY | Country of residence |
| CD_PAT_NURS_HOM | Does the patient reside in a nursing home? (coding: 0 = No, 1 = Yes, 9 = No answer) |
| CD_SITE | Campus establishment number, pseudonymized, with postal code of the site for spatial analyses |
| TX_DATA_PROV_VAL | RIZIV code of the data provider institution, pseudonymized, with postal code of the institution for spatial analyses |
| CD_PAT_ADDED | Was this patient added to the sample to reach the minimum of 25 type 1 diabetics? |
| CD_DIABT_TPE | Type of diabetes (coding: 1 = T1D, 2 = T2D, 3 = Other) |
| DT_DIAGS_DIABT | Date of diabetes diagnosis, coded as exact relative date relative to 01 January of year of birth AND absolute diagnosis date reduced to month level (01/month/year) |
| DT_START_TREAT_ORL_ANTDIA | Start date of treatment with oral antidiabetics, coded as exact relative date relative to 01 January of year of birth AND absolute date reduced to month level (01/month/year) |
| DT_START_TREAT_INSUL | Start date of treatment with insulin, coded as exact relative date relative to 01 January of year of birth AND absolute date reduced to month level (01/month/year) |
| DT_PAT_ADD_CONVT | Inclusion date of the patient in your convention, coded as exact relative date relative to 01 January of year of birth AND absolute date reduced to month level (01/month/year) |
| CD_PAT_TRSPLT_DIALYSIS | Has the patient already had a kidney transplant or is he/she undergoing hemodialysis or peritoneal dialysis? (coding: 0 = No, 1 = Yes, 9 = No answer) |
| CD_PAT_LASR_RET | Has the patient ever received treatment (laser photocoagulation and/or intravitreal injection) for diabetic retinopathy? (coding: 0 = No, 1 = Yes, 9 = No answer) |
| CD_PAT_LASR_RET_AUDIT | If yes, during the audit period? (coding: 0 = No, 1 = Yes, 9 = No answer) |
| CD_PAT_LASR_MACL | Has the patient ever received treatment (laser photocoagulation and/or intravitreal injection) for diabetic maculopathy? (coding: 0 = No, 1 = Yes, 9 = No answer) |
| CD_PAT_LASR_MACL_AUDIT | If yes, during the audit period? (coding: 0 = No, 1 = Yes, 9 = No answer) |
| CD_PAT_DIABT_RET | Does the patient have diabetic retinopathy? (coding: 0 = No, 1 = Yes, 9 = No answer) |
| CD_PAT_BLIND | Is the patient blind? (coding: 0 = No, 1 = Yes, 9 = No answer) |
| CD_PAT_DIABT_FT_WND | Has the patient ever had a diabetic foot ulcer? (coding: 0 = No, 1 = Yes, 9 = No answer) |
| CD_PAT_DIABT_FT_WND_AUDIT | If yes, during the audit period? (coding: 0 = No, 1 = Yes, 9 = No answer) |
| CD_PAT_REVASC | Has the patient ever undergone revascularization of the lower limbs? (coding: 0 = No, 1 = Yes, 9 = No answer) |
| CD_PAT_REVASC_AUDIT | If yes, during the audit period? (coding: 0 = No, 1 = Yes, 9 = No answer) |
| CD_PAT_MINR_AMP | Has the patient ever had a minor amputation of the lower limbs? (coding: 0 = No, 1 = Yes, 9 = No answer) |
| CD_PAT_MAJR_AMP | Has the patient ever had a major amputation of the lower limbs? (coding: 0 = No, 1 = Yes, 9 = No answer) |
| CD_PAT_MI | Has the patient ever had a myocardial infarction? (coding: 0 = No, 1 = Yes, 9 = No answer) |
| CD_PAT_MI_AUDIT | If yes, during the audit period? (coding: 0 = No, 1 = Yes, 9 = No answer) |
| CD_PAT_PCI | Has the patient ever had a PCI (Percutaneous Coronary Intervention)? (coding: 0 = No, 1 = Yes, 9 = No answer) |
| CD_PAT_PCI_AUDIT | If yes, during the audit period? (coding: 0 = No, 1 = Yes, 9 = No answer) |
| CD_PAT_CABG | Has the patient ever had a CABG (Coronary Artery Bypass Graft)? (coding: 0 = No, 1 = Yes, 9 = No answer) |
| CD_PAT_CABG_AUDIT | If yes, during the audit period? (coding: 0 = No, 1 = Yes, 9 = No answer) |
| CD_PAT_STROKE | Has the patient ever had a cerebrovascular accident (stroke)? (coding: 0 = No, 1 = Yes, 9 = No answer) |
| CD_PAT_STROKE_AUDIT | If yes, during the audit period? (coding: 0 = No, 1 = Yes, 9 = No answer) |
| CD_PAT_TCAR | Has the patient ever had a revascularization of the carotid arteries? (coding: 0 = No, 1 = Yes, 9 = No answer) |
| CD_PAT_TCAR_AUDIT | If yes, during the audit period? (coding: 0 = No, 1 = Yes, 9 = No answer) |
| CD_PAT_PCI_CABG | Has the patient ever had a coronary revascularization (PCI/CABG)? (coding: 0 = No, 1 = Yes, 9 = No answer) |
| CD_PAT_PCI_CABG_AUDIT | If yes, during the audit period? (coding: 0 = No, 1 = Yes, 9 = No answer) |
| CD_PAT_HFAE_DIAGS | Does the patient have a diagnosis of heart failure confirmed and monitored by a cardiologist? (coding: 0 = No, 1 = Yes, 9 = No answer) |
| CD_PAT_MSMENT | The patient uses the following glucose monitoring method (coding: fingerstick_test = Finger prick test, Sensor-based continuous glucose monitoring (rtCGM/isCGM), no_response = No answer) |
| CD_PAT_STA_SMOK | Smoking status |
| MS_BMI | BMI (kg/m²), calculated by the IKED coordinator based on MS_PAT_LEN and MS_PAT_WGHT (not the one calculated in HD4DP form) |
| MS_WAIST_CIRCUM | Waist circumference (cm) |
| MS_SYS_BLD_PRESR | Systolic blood pressure (mmHg) |
| MS_DIA_BLD_PRESR | Diastolic blood pressure (mmHg) |
| CD_HBA1C | Was HbA1c measured? (coding: 0 = No, 1 = Yes) |
| MS_HBA1C | HbA1c (%) |
| MS_TIR_14D | Time in Range (TIR) over a period of 14 days (%) – (70–180 mg/dl) |
| MS_TIR_14D_LEVEL1 | Time Below Range (TBR) over a period of 14 days (%) – Level 1 (54–69 mg/dl) |
| MS_TIR_14D_LEVEL2 | Time Below Range (TBR) over a period of 14 days (%) – Level 2 (<54 mg/dl) |
| MS_COEFF_VAR_14D | Coefficient of Variation over a period of 14 days (%) |
| CD_BLD_LIP | Were blood lipids measured? (coding: 0 = No, 1 = Yes) |
| MS_BLD_LIP_CHOL | Total cholesterol (mg/dl) |
| MS_BLD_LIP_HDL_CHOL | HDL cholesterol (mg/dl) |
| MS_BLD_LIP_TRIG | Triglycerides (mg/dl) |
| CD_BLD_LIP_PAT_SOB | Was the patient fasting? (coding: 0 = No, 1 = Yes) |
| MS_BLD_LIP_LDL_LAB | LDL cholesterol (mg/dl) measured in the lab |
| CD_SERM_CREAN | Was serum creatinine measured? (coding: 0 = No, 1 = Yes) |
| MS_SERM_CREAN | Serum creatinine (mg/dl) |
| CD_PAT_SCRNG_MCRALB | Was microalbuminuria measured? (coding: 0 = No, 1 = Yes) |
| MS_VAL_MCRALB | Value for albuminuria |
| CD_MCRALB_UNT | Specify the unit in which albuminuria is expressed |
| CD_SCRNG_RET | Was a screening for retinopathy performed? |
| CD_SENS_TEST_BIOTH | Was a sensitivity test performed (monofilament, biothesiometer, or tuning fork)? (coding: 0 = No, 1 = Yes) |
| CD_SENS_TEST_BIOTH_RESULT | Result of the sensitivity test (coding: 1 = Normal, 2 = Abnormal, 9 = No answer) |
| CD_EXAM_FT_PULSE | Was an examination of foot pulses performed? (coding: 0 = No, 1 = Yes) |
| CD_EXAM_FT_PULSE_RESULT | Result of foot pulse examination (coding: 1 = Pulses present, 2 = Pulses absent, 9 = No answer) |
| CD_PAT_HAD_SEV_HYPO | Has the patient had episodes of SEVERE hypoglycemia? (coding: 0 = No, 1 = Yes, 9 = No answer) |
| CD_PAT_DKA | Was the patient hospitalized for ketoacidosis or for a hyperosmolar hyperglycemic state (with or without ketosis)? (coding: 0 = No, 1 = Yes, 9 = No answer) |
| CD_INSUL_SCHM | Insulin regimen (coding: 4 = Insulin pump, 6 = Injections, 9 = No answer) |
| CD_CLOSE_LOOP_SYSTM | If using pump and sensor: does the patient use a (hybrid) closed-loop system? (coding: 0 = No, 1 = Yes, 9 = No answer) |
| CD_TREAT_BIGU | Treatment with biguanides? (coding: 0 = No, 1 = Yes, 9 = No answer) |
| CD_TREAT_SULF | Treatment with sulfonylureas and/or glinides? (coding: 0 = No, 1 = Yes, 9 = No answer) |
| CD_TREAT_GLIT | Treatment with glitazones? (coding: 0 = No, 1 = Yes, 9 = No answer) |
| CD_TREAT_DPP4_INHIB | Treatment with DPP-4 inhibitors? (coding: 0 = No, 1 = Yes, 9 = No answer) |
| CD_TREAT_SGLT2_INHIB | Treatment with SGLT2 inhibitors? (coding: 0 = No, 1 = Yes, 9 = No answer) |
| CD_TREAT_INCRET_MIMET | Treatment with incretin mimetics or GLP-1 receptor agonists? (coding: 0 = No, 1 = Yes, 9 = No answer) |
| CD_TREAT_STATN | Treatment with statins? (coding: 0 = No, 1 = Yes, 9 = No answer) |
| CD_TREAT_FIBRT | Treatment with fibrates? (coding: 0 = No, 1 = Yes, 9 = No answer) |
| CD_TREAT_EZE | Treatment with ezetimibe? (coding: 0 = No, 1 = Yes, 9 = No answer) |
| CD_TREAT_OTH_LIP_LOW_MEDICT | Treatment with other lipid-lowering medication? (coding: 0 = No, 1 = Yes, 9 = No answer) |
| CD_TREAT_AGG_INHIB | Treatment with antiplatelet agents or anticoagulants? (coding: 0 = No, 1 = Yes, 9 = No answer) |
| CD_TREAT_ACE_INHIB | Treatment with ACE inhibitors or sartans? (coding: 0 = No, 1 = Yes, 9 = No answer) |
| CD_PAT_OTH_HTN | Does the patient receive other antihypertensive drugs? (coding: 0 = No, 1 = Yes, 9 = No answer) |
| CD_PAT_NEUROPT_PAIN | Is the patient treated for neuropathic pain? (coding: 0 = No, 1 = Yes, 9 = No answer) |

**APPENDIX LIST OF SELECTED VARIABLES IN IQED**

A detailed description of the dataset can be found online: <https://fair.healthdata.be/dataset/01e61048-4f6c-464c-b01e-7d3fb613530c>

All patients registered in IQECAD are diabetes patients and are selected as such. Based on the two personal lists drawn up from IQE(CA)D and Intego respectively, a third unique personal list is compiled which will be linked to IMA. In this new unique personal list, it will be indicated whether a patient is found in the IQE(CA)D database and/or the Intego database.

For the selected patients, the following data are required:

| **Variabele** | **Description** |
| --- | --- |
| IDC_PAT | Double-coded national registry number of the patient |
| DT_START_AUDIT | Start date of the IQECAD audit period |
| DT_END_AUDIT | End date of the IQECAD audit period |
| Nr_path_birthy | Year of birth |
| Nr_path_deathy | Year of death |
| CD_PAT_SEX | Sex (male/female/unknown) |
| CD_PAT_NIS | Place of residence of the patient, at district level |
| CD_CNTRY | Country of residence |
| CD_DATA_PROV_VAL | RIZIV code of the data provider institution, pseudonymized, with postal code of the institution for spatial analyses |
| CD_PAT_SAMPLING | Was this patient selected during step 1 or step 2 of the sampling? (coding: 1 = Selected during step 1, 2 = Selected during step 2) |
| CD_TREAT_PAT_INI_PATH | Is this patient treated in a center in the introduction trajectory? (coding: 0 = No, 1 = Yes) |
| CD_DIABT_TPE | Type of diabetes (coding: 1 = T1D, 2 = T2D, 3 = Other) |
| DT_DIAGS_DIABT | Date of diabetes diagnosis, coded as exact relative date relative to 01 January of year of birth AND absolute diagnosis date reduced to month level (01/month/year) |
| DT_START_TREAT_INSUL | Start date of insulin treatment, coded as exact relative date relative to 01 January of year of birth AND absolute date reduced to month level (01/month/year) |
| DT_PAT_ADD_CONVT | Date of patient inclusion in your convention, coded as exact relative date relative to 01 January of year of birth |
| CD_PAT_MSMENT_METH | The patient uses the following glucose monitoring method (coding: fingerstick_test = Finger prick test, continuous_glucose_monitoring_cgm = Continuous Glucose Monitoring (CGM), flash_glucose_monitoring_fgm = Flash Glucose Monitoring (FGM), no_response = No answer) |
| CD_GCM_PAT_INCL_CONVT | Is the patient included in the CGM convention? (coding: 0 = No, 1 = Yes) |
| MS_FGM_SCAN_DAY | Average number of scans per day |
| MS_CVG_14D_FGM | For FGM use: What was the average time coverage during a 14-day period (% time coverage)? |
| MS_RNG_14D_FGM | For FGM use: What was the average time in range (between 70 and 180 mg/dl) during a 14-day period (% time in range)? |
| MS_HYPO_14D_FGM | For FGM use: What was the average percentage of time spent in hypoglycemia (<70 mg/dl) during a 14-day period (%)? |
| MS_CVG_14D_CGM | For CGM use: What was the average time coverage during a 14-day period (% time coverage)? |
| MS_RNG_14D_CGM | For CGM use: What was the average time in range (between 70 and 180 mg/dl) during a 14-day period (% time in range)? |
| MS_HYPO_14D_CGM | For CGM use: What was the average percentage of time spent in hypoglycemia (<70 mg/dl) during a 14-day period (%)? |
| MS_MSMENT_GLU_BLD_DAY | Average number of glucose measurements per day |
| MS_BMI_NEW | BMI recalculated based on validated weight and height. Not the BMI automatically calculated in the HD4DP form (= ms_bmi). |
| MS_SYS_BLD_PRESR | Systolic blood pressure (mmHg) |
| MS_DIA_BLD_PRESR | Diastolic blood pressure (mmHg) |
| CD_HBA1C | Was HbA1c measured? (coding: 0 = No, 1 = Yes) |
| MS_HBA1C_PCT | HbA1c (%) – 3 or more items |
| DT_HBA1C_PCT | Date of HbA1c measurement, coded as exact relative date relative to 01 January of year of birth. |
| MS_HBA1C_NUM | Number of HbA1c measurements |
| CD_BLD_LIP | Were blood lipids measured? (coding: 0 = No, 1 = Yes) |
| MS_BLD_LIP_CHOL | Total cholesterol (mg/dl) |
| MS_BLD_LIP_HDL_CHOL | HDL cholesterol (mg/dl) |
| MS_BLD_LIP_TRIG | Triglycerides (mg/dl) |
| MS_BLD_LIP_LDL_CHOL | LDL cholesterol, calculated (mg/dl) |
| CD_BLD_LIP_PAT_FAST | Was the patient fasting? (coding: 0 = No, 1 = Yes) |
| MS_BLD_LIP_LDL_LAB | LDL cholesterol (mg/dl) measured in the lab |
| CD_MICROALBUMINURIA | Was microalbuminuria measured? |
| CD_NEPHRO_SCRNG_RECEIVD | Has the patient already had a screening for nephropathy? (coding: 0 = No, 1 = Yes) |
| CD_PAT_MCRALB | Does the patient have (micro)albuminuria? |
| CD_NEPHRO_LAST_SCRNG_POS | Was the last screening positive? (coding: 0 = No, 1 = Yes, 9 = No answer) |
| CD_NEPHRO_LAST_SCRNG_CUR_YR | Did the last screening take place during the audit year? (coding: 0 = No, 1 = Yes) |
| CD_RETINOPATHY | Was a screening for retinopathy performed? |
| CD_RETINO_SCRNG_RECEIVD | Has the patient already had a screening for retinopathy? (coding: 0 = No, 1 = Yes) |
| CD_PAT_DIABT_RET | Does the patient have diabetic retinopathy? |
| CD_RETINO_LAST_SCRNG_POS | Was the last screening positive? (coding: 0 = No, 1 = Yes, 9 = No answer) |
| CD_RETINO_LAST_SCRNG_CUR_YR | Did the last screening take place during the audit year? (coding: 0 = No, 1 = Yes) |
| CD_PAT_HAD_SEV_HYPO | Has the patient had episodes of SEVERE hypoglycemia? (coding: 0 = No, 1 = Yes, 9 = No answer) |
| MS_PAT_NR_SEV_HYPO | If yes, how many episodes of severe hypoglycemia WITHIN A PERIOD OF 3 MONTHS? |
| CD_ADMI_DKA_HYPRGL_ADT | Was the patient admitted for ketoacidosis or for a hyperosmolar hyperglycemic state (with or without ketosis) during the audit year? (coding: 0 = No, 1 = Yes, 9 = No answer) |
| CD_PAT_DKA | Was the patient admitted for ketoacidosis or for hyperosmolar hyperglycemia with or without ketosis? |
| MS_PAT_NR_DKA_ADMIS | If yes, how many admissions? |
| CD_CELIAC | Was a screening for celiac disease performed? |
| CD_CELIAC_SCRNG_RECEIVD | Has the patient already had a screening for celiac disease? (coding: 0 = No, 1 = Yes) |
| CD_CELIAC_RESULT | Was the result of the celiac disease screening positive? |
| CD_CELIAC_LAST_SCRNG_POS | Was the last screening positive? (coding: 0 = No, 1 = Yes, 9 = No answer) |
| CD_CELIAC_LAST_SCRNG_CUR_YR | Did the last screening take place during the audit year? (coding: 0 = No, 1 = Yes) |
| CD_THYROID_AUTIM | Was a screening for thyroid autoimmunity performed? |
| CD_THYROID_SCRNG_RECEIVD | Has the patient already had a screening for thyroid autoimmunity? (coding: 0 = No, 1 = Yes) |
| CD_THYROID_AUTIM_POS | Was the result of the thyroid autoimmunity test positive? |
| CD_THYROID_LAST_SCRNG_POS | Was the last screening positive? (coding: 0 = No, 1 = Yes, 9 = No answer) |
| CD_THYROID_LAST_SCRNG_CUR_YR | Did the last screening take place during the audit year? (coding: 0 = No, 1 = Yes) |
| CD_NEUPAT | Was a screening for neuropathy performed? |
| CD_NEURO_SCRNG_RECEIVD | Has the patient already had a screening for neuropathy? (coding: 0 = No, 1 = Yes) |
| CD_NEURO_LAST_SCRNG_POS | Was the last screening positive? (coding: 0 = No, 1 = Yes, 9 = No answer) |
| CD_NEURO_LAST_SCRNG_CUR_YR | Did the last screening take place during the audit year? (coding: 0 = No, 1 = Yes) |
| CD_INSUL_SCHM | Insulin regimen (coding: 1 = ≤2 injections per day, 2 = 3 injections per day, 3 = 4 or more injections per day, 4 = Insulin pump, 5 = Other regimen, 9 = No answer) |
| CD_TPE_TECH_MAIN_USE | If “Insulin pump”: Which type of technology did the patient mainly use during the audit year? (coding: Pump without sensor feedback, Sensor-augmented pump (SAP), Hybrid closed-loop pumps, Pump with fingerstick tests) |
| MS_INSUL_DOS_DAY | Insulin dose (IU/day) |
| CD_PAT_TREAT_CELIAC | Is the patient treated for celiac disease? (coding: 0 = No, 1 = Yes, 9 = No answer) |
| CD_PAT_TREAT_HYPOT_HYPRT | Is the patient treated for hypo- or hyperthyroidism? (coding: 0 = No, 1 = Yes, 9 = No answer) |
| CD_PAT_TREAT_EPIL | Is the patient treated for epilepsy? (coding: 0 = No, 1 = Yes, 9 = No answer) |
| CD_PAT_TREAT_ARTER_HTN | Is the patient treated for arterial hypertension? (coding: 0 = No, 1 = Yes, 9 = No answer) |
| CD_PAT_TREAT_DYSLIP | Is the patient treated for dyslipidemia? (coding: 0 = No, 1 = Yes, 9 = No answer) |
| MS_CONSTTN_DIABT_CENT | Number of consultations in the diabetes center |

**APPENDIX LIST OF SELECTED VARIABLES IN INTEGO**

A detailed description of the dataset can be found online: <https://fair.healthdata.be/dataset/7bc34c8c-40d1-483f-8da5-2a4b6b0aaace>

Diabetes patients registered in Intego are identified based on:

- The International Classification of Primary Care (ICPC) code assigned by the general practitioner (T89; T90; W85)
- And/or registration of follow-up within a diabetes care trajectory

Based on the two personal lists drawn up from IQE(CA)D and Intego respectively, a third unique personal list is compiled which will be linked to IMA. In this new unique personal list, it will be indicated whether a patient is found in the IQE(CA)D database and/or the Intego database.

For the selected patients, the following data are required:

| **Variabele** | **Description** |
| --- | --- |
| ***PATIENT MASTER*** |  |
| PATIENT_ID | Double-coded national registry number of the patient |
| BIRTH_YEAR | Year of birth |
| DEATH_DATE | Year of death |
| GENDER | Sex (coding: F = Female, M = Male, U = Unknown) |
| ***PATIENT DYNAMIC*** |  |
| PATIENT_ID | Double-coded national registry number of the patient |
| POSTAL_CODE | Place of residence, postal code |
| PRACTICE_ID | Practice identification, pseudonymized |
| ***DIAGNOSES*** |  |
| PATIENT_ID | Double-coded national registry number of the patient |
| ICPC2 | icpc2Code |
| ICD10 | icd10Code |
| IBUI | ibuiCode |
| DIAG_TEXT | Care approach Title |
| DATE (ABSOLUTE & RELATIVE) | Date of diagnosis, coded as exact relative date relative to 01 January of year of birth AND absolute date reduced to month level (01/month/year) |
| ***HISTORY CHRONIC DISEASES*** |  |
| PATIENT_ID | Double-coded national registry number of the patient |
| ICPC2 | icpc2Code |
| ICD10 | icd10Code |
| IBUI | ibuiCode |
| DIAG_TEXT | Care approach Title |
| DATE (ABSOLUTE & RELATIVE) | Start date of care element, coded as exact relative date relative to 01 January of year of birth AND absolute date reduced to month level (01/month/year) |
| ***LABORATORY RESULTS*** |  |
| PATIENT_ID | Double-coded national registry number of the patient |
| PRACTICE_ID | Practice identification, pseudonymized |
| MEDIDOC_CODE | Medidoc code |
| LAB_RESULT (CLEAN_RESULT) | Result |
| LAB_UNIT | Unit |
| REF_RANGE | Reference value |
| DATE (ABSOLUTE & RELATIVE) | Date of laboratory test request, coded as exact relative date relative to 01 January of year of birth AND absolute date reduced to month level (01/month/year) |
| ***CARE APPROACHES*** |  |
| PATIENT_ID | Double-coded national registry number of the patient |
| APPROACH_ID | Care approach title (coding: 1 = Pre-traject type 2 diabetes, 2 = Care trajectory chronic renal insufficiency, 3 = Care trajectory type II diabetes) |
| START_DATE (ABSOLUTE & RELATIVE) | Care approach start date, coded as exact relative date relative to 01 January of year of birth AND absolute date reduced to month level (01/month/year) |
| END_DATE (ABSOLUTE & RELATIVE) | Care approach end date, coded as exact relative date relative to 01 January of year of birth AND absolute date reduced to month level (01/month/year) |
| ***MEASUREMENTS*** |  |
| PATIENT_ID | Double-coded national registry number of the patient |
| MEASURE_CODE | Parameter type (coding:  4 – Diastolic blood pressure  5 – Systolic blood pressure  6 – Heart rate  7 – Rhythm  8 – Temperature  10 – Oxygen saturation  14 – FEV1  15 – GOLD classification  25 – Ejection fraction) |
| MEASURE_VALUE | Value |
| MEASURE_UNIT | Unit |
| DATE (ABSOLUTE & RELATIVE) | Date, coded as exact relative date relative to 01 January of year of birth AND absolute date reduced to month level (01/month/year) |
| ***PATIENT LIFESTYLE*** |  |
| PATIENT_ID | Double-coded national registry number of the patient |
| PHYSICAL_ACTIVITY, SMOKING_STATUS, ALCOHOL_USE | Parameter type (11 = Physical activity, 12 = Smoking status, 13 = Alcohol use) |
| DATE (ABSOLUTE & RELATIVE) | Date, coded as exact relative date relative to 01 January of year of birth AND absolute date reduced to month level (01/month/year) |
| ***PATIENT BODY MEASURES*** |  |
| PATIENT_ID | Double-coded national registry number of the patient |
| LENGTH_CM, WEIGHT_KG, BMI, ABD_CIRCUM | Parameter type (coding: 1 = height, 2 = weight, 3 = BMI, 9 = waist circumference) |
| DATE (ABSOLUTE & RELATIVE) | Date, coded as exact relative date relative to 01 January of year of birth AND absolute date reduced to month level (01/month/year) |
| ***HISTORY INTOLERANCE*** |  |
| PATIENT_ID | Double-coded national registry number of the patient |
| MEDINTOL_TEXT | Packaging |
| DATE (ABSOLUTE & RELATIVE) | Date of medication intolerance, coded as exact relative date relative to 01 January of year of birth AND absolute date reduced to month level (01/month/year) |
| ***PRESCRIPTIONS*** |  |
| PATIENT_ID | Double-coded national registry number of the patient |
| PRES_ATC | ATC code |
| PRES_CNK | Identification number |
| PRES_STATUS | Status (coding: 1 = active; 2 = passive) |
| START_DATE (ABSOLUTE & RELATIVE) | Start date of medication use, coded as exact relative date relative to 01 January of year of birth AND absolute date reduced to month level (01/month/year) |
| END_DATE (ABSOLUTE & RELATIVE) | End date of medication use, coded as exact relative date relative to 01 January of year of birth AND absolute date reduced to month level (01/month/year) |
| PRES_TYPE | Usage period (coding: 1 = chronic; 2 = acute; 3 = one-time; 4 = as needed) |
| DOSAGE_TEXT | Dosage |
| ***HISTORY PROCEDURES*** |  |
| PATIENT_ID | Double-coded national registry number of the patient |
| PROCED_TEXT |  |
| DATE (ABSOLUTE & RELATIVE) | Date of procedure, coded as exact relative date relative to 01 January of year of birth AND absolute date reduced to month level (01/month/year) |

**APPENDIX LIST OF SELECTED VARIABLES IN IMA**

A detailed description of the dataset can be found online: https://metadata.ima-aim.be/nl/

For the selected patients, the following data are required:

| **Variabele** | **Description** |
| --- | --- |
| PP0010 (DATA GROUP POPULATION), SS00010 (DATA GROUP HEALTHCARE AND FARMANET). | Double-coded national registry number of the patient |
| ***DATA GROUP POPULATION*** |  |
| PP0015A | Year of birth |
| PP0020 | Gender (coding: 1 = male, 2 = female) |
| PP0040A | Year of death |
| PP0025 | Place of residence (NIS–5 code, municipality) |
| ***DATA GROUP HEALTHCARE*** |  |
| PROCEDURE_AH_CAT | Distinction between outpatient and hospitalized The variable indicates whether the nomenclature code (SS00020) relates to a service provided for a non-hospitalized patient (outpatient care) or for a hospitalized patient. |
| ATC_PROD_L | ATC code level 5 |
| C_RANK | Service day sequence number The sequence number, per beneficiary and per service year, of the day on which care was provided. This information is derived from the start date of the service (including positive, negative, and zero expenditures). |
| SS00015 | Start date of service, coded as an exact relative date with respect to January 1 of the birth year AND absolute date reduced to the month level (01/month/year) The date on which the care service was provided.  If the nomenclature code refers to a series of services (e.g., a series of nine physiotherapy sessions), the date refers to the first in the series. The number of services in this series can be found in Number of cases.  In the case of hospital admission or stay, the variable refers to the first billed day of the billing period. The number of days associated with the nomenclature code for this stay can be found in Number of days.For pharmaceutical products (SS00135 ≠ 0), the variable indicates the administration date or the start date of the administration period. |
| SS00020 | Nomenclature code |
| SS00050 | Number of cases The number of times a service was provided or the number of deliveries (e.g., blood, isotopes…) on the specified date or during the specified period (SS00015–SS00125).  For a pharmaceutical product, the variable refers to the number of units delivered on the specified date or during the specified period. This corresponds to the number of times the unit fee was charged. |
| SS00055 | Number of days Number of billed days, flat rates, or leave days |
| SS00060_100P | Nursing day rate at 100% |
| SS00060 | ZIV amount |
| PRACTITIONER_CAT | Professional code of the healthcare provider performing the service |
| SS00065B | Qualification healthcare provider |
| PRESCRIBER_CAT | Professional code of the healthcare provider |
| SS00070B | Qualitificaiton prescriber |
| INSTITUTION_CAT | Type of institution The type of institution where the patient resides. This is a code derived from the first three digits of the institution’s identification number. |
| SS00075 | Institution identification number, coded with NIS-5 code (municipality) |
| SS00080 | Service code |
| INSTITUTION_SITE_CAT | The type of facility where the service was actually provided |
| SS00085 | Place of service, coded with NIS-5 code (municipality) |
| SS00125 | Date of last service, coded as an exact relative date with respect to January 1 of the birth year AND absolute date reduced to month level (01/month/year) |
| SS00135 | CNK code |
| SS00155 | Prescription date, coded as an exact relative date with respect to January 1 of the birth year AND absolute date reduced to month level (01/month/year) |
| SS00160 | Patient co-payment |
| SS00165 | Supplement |
| SS00355 | Galilean form of magistral preparation |
| ***DATA GROUP HOSPITALISATIONS*** | The Hospitalizations database contains data on hospital stays recorded by the seven Belgian health insurance funds. Variables are derived from the Healthcare database. Data are provided per calendar year. |
| ADMISSION | Admission date, coded as exact relative date with respect to January 1 of birth year AND absolute date reduced to month level (01/month/year) |
| LOS | Length of stay |
| SERV_ADM | Admission department |
| STAY_CAT | Type of admission/stay |
| STAY_NR | Year/sequence number of admissions/stays |
| ***DATA GROUP***  ***FARMANET*** | The Farmanet database contains information on reimbursed medicines and medical devices delivered in public pharmacies. |
| PROCEDURE_AH_CAT | Distinction between outpatient and hospitalized This variable indicates whether the nomenclature code (SS00020) relates to a service provided for a non-hospitalized patient or for a hospitalized patient. Since Farmanet only contains pharmaceutical products delivered by public pharmacies, the value of this variable is always 1 (Outpatient). |
| ATC_PROD_L | ATC code level 5 |
| SS00015 | Delivery date, coded as exact relative date with respect to January 1 of birth year AND absolute date reduced to month level (01/month/year) The date on which the medicine was delivered to the beneficiary. If it is a fee, reimbursement, or other service (identifiable by pseudonomenclature code), it is the date on which it was charged or started. |
| SS00020 | Pseudonomenclature code category of medicine This pseudonomenclature code usually indicates the reimbursement category of the medicine. In other cases, it may indicate whether it is a magistral preparation, fee, or flat rate. |
| SS00050 | Number of cases The number of packages, modules, units, flat rates, etc., delivered to a beneficiary in a public pharmacy. General rule: for pharmaceutical specialties (medicines produced by the pharmaceutical industry), each package is recorded as a separate observation. The quantity for each observation is 1. Exceptions to this rule are noted separately. You can calculate the number of DDD (Defined Daily Dose) based on this variable. |
| SS00060 | ZIV amount |
| SS00070B | Prescriber qualification |
| SS00075 | Institution identification number, coded with NIS-5 code (municipality) |
| INSTITUTION_CAT | Type of residential institution The type of nursing home (RVT-ROB) where the patient resides. This is a code derived from the first three digits of the institution’s identification number. |
| SS00080 | Galilean form of magistral preparation |
| SS00130 | Magistral preparation key |
| SS00135 | CNK code The CNK code is a unique identification number per package form assigned to all medicines and para-pharmaceutical products (medical devices, supplements, cosmetics, etc.) delivered in pharmacies. The assignment is done by APB (General Pharmaceutical Association) in close cooperation with RIZIV for reimbursable medicines. |
| SS00140 | 1st unit position |
| SS00145 | 2nd unit position |
| SS00155 | Prescription date, coded as exact relative date with respect to January 1 of birth year AND absolute date reduced to month level (01/month/year) |
| SS00160 | Patient co-payment |
| SS00165 | Reduction of reimbursement amount |
| SS00195 | Pharmacy fee |
| SS00210 | Supplement |
| SS00430 | Pharmacist fee – ZIV The part of the pharmacist’s fee reimbursed by health insurance. This is an informational amount not invoiced as such but recorded for statistical purposes. |
| SS00435 | Pharmacist fee – Patient The part of the pharmacist’s fee that belongs to the patient’s personal share (co-payment). This is an informational amount not invoiced as such but recorded for statistical purposes. |
| PROCEDURE_GROUP | Cost group |
